# The Structural and Mechanical Properties of the UHMWPE Films Mixed with the PE-Wax

**DOI:** 10.3390/ma13153422

**Published:** 2020-08-03

**Authors:** Tarek Dayyoub, Leonid K. Olifirov, Dilyus I. Chukov, Sergey D. Kaloshkin, Evgeniy Kolesnikov, Saidkhodzha Nematulloev

**Affiliations:** Department of Physical Chemistry, National University of Science and Technology “MISIS”, 119049 Moscow, Russia; leonidolifirov@gmail.com (L.K.O.); dil_chukov@mail.ru (D.I.C.); kaloshkin@misis.ru (S.D.K.); kea.misis@gmail.com (E.K.); nematulloev_said@mail.ru (S.N.)

**Keywords:** UHMWPE, PE-wax, lamellar structure, films, crystallization, mechanical and tribological properties, work of fracture, cavitation phenomena

## Abstract

Since obtaining a highly oriented structure based on a large-scale commercial ultra-high molecular weight polyethylene (UHMWPE) is considered very difficult due to its high molecular weight and melting index, modifying the structure of these cheap commercial UHMWPE brands into a supra-molecular structure with fiber-forming properties by adding a small amount of polyethylene wax (PE-wax) will provide the possibility to obtain highly oriented UHMWPE products with enhanced mechanical and tribological properties. In this work, highly oriented UHMWPE/PE-wax films were prepared. The PE-wax affected the UHMWPE as an intermolecular lubricant. The obtained lamellar structure of the UHMWPE/PE-wax composites had a better processability. The UHMWPE and UHMWPE/PE-wax structures for the xerogels and the films were studied by using differential scanning calorimetry and scanning electron microscopy. The PE-wax presence enhanced the mechanical properties of the UHMWPE/PE-wax films to a high degree. The highest average value of the tensile strength was 1320 MPa (an increase of 78%) obtained by adding a PE-wax content of 1.0 wt.%, and the highest average value of the Young’s modulus was 56.8 GPa (an increase of 71%) obtained by adding a PE-wax content of 2.0 wt.%. The addition of the PE-wax increased the work of fracture values of the UHMWPE/PE-wax films up to 233%. The formation of the cavities was observed in the virgin UHMWPE films more than in the UHMWPE/PE-wax films, and the whitening of the oriented films was related to the crystallization process more than to the cavitation phenomenon. The coefficient of friction of the oriented UHMWPE/PE-wax films improved by 33% in comparison with the isotropic UHMWPE, and by 7% in comparison with the oriented virgin UHMWPE films.

## 1. Introduction

Among polymeric materials, ultrahigh molecular weight polyethylene (UHMWPE) is considered the best choice for medical and light-weight engineering applications in the construction and automotive industries, since it exhibits a unique toughness, abrasion, fatigue, and good chemical resistances, in addition to a very low coefficient of friction, strength, and biocompatibility [1,2,3,4,5]. Polyethylene chains have a covalent C-C bond with a very high energy of 335 kJ/mol. Therefore, obtaining materials based on UHMWPE with an oriented supra-molecular structure and an enhanced mechanical performance is considered an important direction in the polymer science. For example, the tensile strength and the modulus of elasticity for the ultra-oriented fibers based on UHMWPE can reach up to 4 GPa and 140 GPa, respectively [6]. Carrying out the orientation process, tensile stresses are applied on the films during the drawing process, that is to say, the stress and the deformation rates will control the orientation process [7]. Thus, the lamellae, at the beginning, will be moved into the drawing direction, then the crystalline blocks will be reformed into so-called microfibrils, which are sheared, and the intra- and inter- microfibrillar tie-molecules are tautened in a further drawing [8]. Accordingly, the crystallization of the UHMWPE will speed up to a high degree and the mechanical properties will increase as a result of the drawing and the thermal orientation process of the amorphous phase, leading to an improvement in the UHMWPE films by increasing the molecular orientation [7,9]. The maximum attainable draw ratio (λ) of the polyethylene is related to the polymer molecular weight (M_w_) [10] and to the polymer concentration in the solvent solution [11].

Since the UHMWPE has high molecular weight, its melt flow index is considered very high. So, the preparation of highly oriented materials by using injection, blow, extrusion molding, or melt spinning technology will be very difficult based on this quality of UHMWPE [12]. This is because of the large number of entanglements exciting in the UHMWPE amorphous phase. They obstruct the movement of the UHMWPE chains during the orientation process, leading to a premature rupture of the UHMWPE macromolecules and preventing the achievement of high degrees of drawing. Hence, the gel-spinning technology is considered a preferable technology in order to obtain highly oriented materials based on the UHMWPE [13,14].

The addition of polyethylene wax (PE-wax) that has a low molecular weight to the UHMWPE matrix is suggested to be very important for improving the drawing process and the mechanical properties through self-enhancement because it will bring down the melt viscosity value and facilitate the melt processing [15]. The ability of PE-wax to act as a solvent for the UHMWPE leads to an improvement in the mechanical and rheological properties of the UHMWPE [9]. In the reference [16], they reported that the PE-wax was crystallized onto the UHMWPE extended-chains via co-crystallization process, which led to a formation of a shish-kebab structure. They confirmed that this co-crystallization in the UHMWPE/PE-wax fiber led to the prevention of any embrittlement or impairment occurrences in the mechanical properties, which can happen as a result of the PE-wax presence as an independent and unconnected component. They also noted that this in situ reinforcement basically helped in improving the mechanical properties of the HDPE as reflected by simultaneously increased Young’s modulus (from 0.81 GPa to 3.77 GPa) and tensile strength (from 27.3 MPa to 134.2 MPa). In the reference [9], they prepared UHMWPE/PE-wax films by adding a large amount of PE-wax up to 80 wt.% as a solvent for solution spinning operations. They prepared their samples by using a compression-molding method at 180 °C and a pressure of 100 bar for 5 min. Then, the samples were drawn isothermally one by one by applying solid-state drawn in oven at a temperature of 100 °C. They reported that an improvement in the rheological properties and in the maximum attainable draw ratio had been obtained when the UHMWPE (M_w_ = 10^6^ g/mol) was blended with 60 wt.% of a linear polyethylene wax (M_w_ = 1000 g/mol). They also found that the maximum tensile strength of the drawn PE-blends had increased from ~1 GPa to ~1.5 GPa (an increase of 50%), and the maximum Young’s modulus of the drawn films had increased more than two times without the removal of the solvent. In the reference [17], they reported that the presence of the HDPE wax with content up to 54 wt.%, served as a processing aid, lowering melt viscosity, while the presence of the UHMWPE with content up to 63 wt.% led to the improvement of the blend properties. Also, they found that a massive polyethylene self-reinforcement had shown at the 32 wt.% UHMWPE content, which reflected by the improved Young’s modulus (from 0.9 GPa to 4.8 GPa) and tensile strength (from 24 MPa to 201 MPa) without impairing the HDPE injection molding.

There are few works that studied the effect of the orientation process of the PE blends on their tribological properties. In the reference [17], they reported that the tailored PE composites had high wear resistance in comparison with the mono-modal UHMWPE. They noted that high UHMWPE content had led to the improvement of wear resistance, stiffness, strength, and toughness. On the other hand, at low UHMWPE content, the lubricating effect of built-in HDPE wax was likely to contribute to wear resistance. In the reference [18], they prepared HDPE/UHMWPE blends by two types. The first one was prepared by oscillatory shear injection molding (OSIM), and the second one was prepared by conventional injection molded (CIM). They reported that the abrasion resistance of both CIM and OSIM blends had improved in comparison with the virgin HDPE. Also, by increasing the UHMWPE content, the wear rate declined for both CIM and OSIM blends. This was related to the integrity of the UHMWPE microstructure, which was retained during polymer blending; and that had led to the preservation of the wear resistance in the PE blend samples.

In this study, bulk oriented UHMWPE/PE-wax films were prepared. Xerogels of virgin UHMWPE and UHMWPE/PE-wax were drawn and oriented. The prepared bulk-oriented UHMWPE/PE-wax films showed an improvement in the tensile and tribological properties in comparison with the virgin UHMWPE films, which could be considered in the design of the composite materials. The UHMWPE films have a high strength, a slippery surface, and a light weight, so that they are considered as an ideal choice for both industrial and biomedical applications.

## 2. Materials and Methods

### 2.1. Materials

UHMWPE GUR 4120 with a molecular weight of 5 × 10^6^ g/mol and polyethylene wax PLWN-3W with a molecular weight of 4 kg/mol were purchased from “Ticona GmbH” (Germany) and “INHIMTEK LLC” (Novokuibyshevsk, Russia) respectively. PE-wax is a high-quality, non-oxidized, non-polar, linear, low molecular weight polyethylene, produced according to the high-temperature destruction process. P-xylene with a ratio of 2.5 mL of a solvent per 1 g of the polymer blend was used as a plasticizer for the UHMWPE composites.

### 2.2. Fabrication of the Films

Mixing UHMWPE and PE-wax powders was done in a high-energy planetary ball mill APF-3 by using steel drums with a volume of 900 mL. The grinding media was steel balls (their diameter is in the range of 7–9.5 mm). In the mixing process, current water was used in order to cool down the drums and control the temperature of the blends at about 60 °C. The average rotation speed was 450 rpm. The total time for mixing was 90 min. The UHMWPE/PE-wax powders with various PE-wax contents (0.1, 0.2, 0.5, 1.0, 1.5, 2.0, 10.0, and 20.0 wt.%) were prepared.

**Figure 1:**
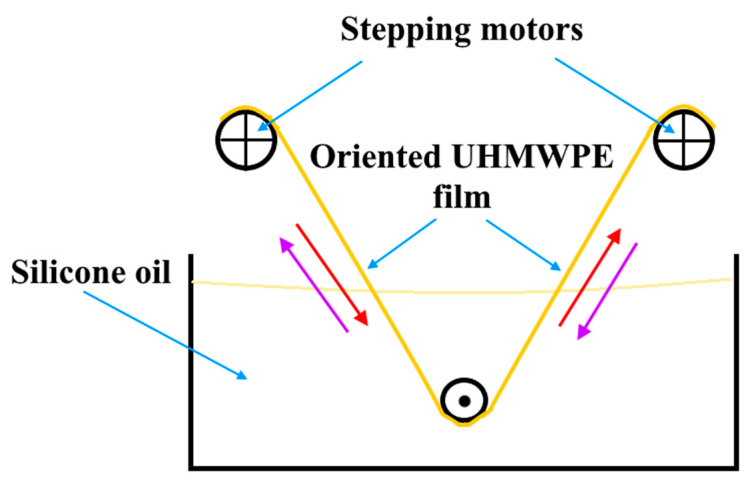
Orientation and drawing process for the UHMWPE films in a silicone oil bath.

Gel-spinning technology was applied to obtain the oriented UHMWPE films by using a small amount of the solvent as a plasticizer in accordance with the approach described in the reference [19]. The first step was mixing the UHMWPE/PE-wax powders and stirring them with p-xylene at 140 ± 2 °C for 20 min. The UHMWPE/p-xylene gel was extruded at a temperature of 150 ± 3 °C by using ram extruder UE-MSL (Extrusion Machinery Sales Ltd); after being stored for 15 min in the extruder at this temperature. The die size was 10 mm × 2 mm and the extrusion rate was equal to 500 mm/min. Then, the obtained gel was dried at room temperature for 2 days to obtain xerogel (solvent-free UHMWPE gel). The primary stage of the thermal orientation of xerogels was carried out by using rolling machine (BL-6175-A). Xerogels were rolled at 110 °C to reach a draw ratio value of 1.5–2.0. In the next stage, a particular laboratory device was used to make the UHMWPE/PE-wax films pass through a bath of a silicone oil and to draw them (Figure 1). The oil temperature was stable within ± 0.1 °C precision. Multi-stages hot orientation process for the UHMWPE films was carried out stepwise at various temperature values ranging from 120 °C to 142 °C. In each stage of the thermal orientation process, each UHMWPE/PE-wax composite had a different range of a draw ratio value at different levels of temperature. 

**Figure 2:**
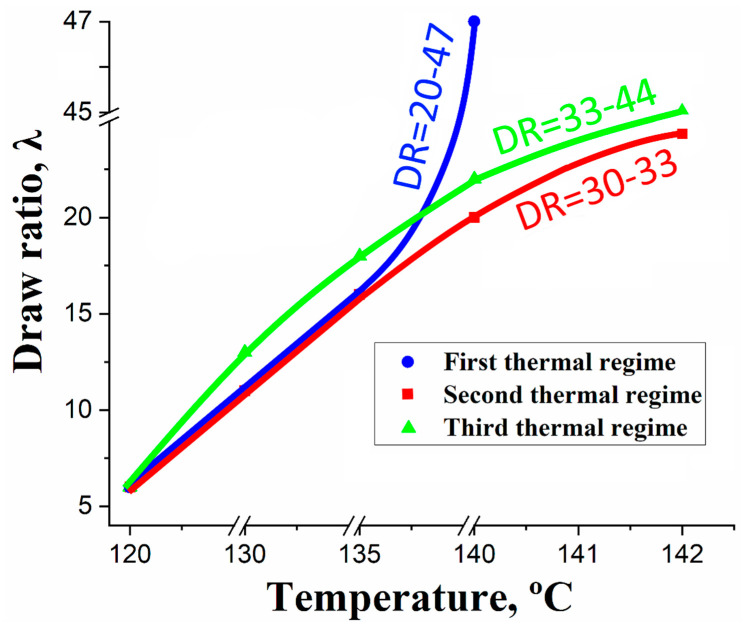
Thermal regimes and obtained draw ratio values in each stage of hot orientation process.

In the orientation process, UHMWPE will be drawn uniaxially by applying tensile stresses at a drawing temperature below its melting temperature, which will lead to the occurrence of a morphological transformation in the UHMWPE structure from a lamellar state to a highly oriented state [9,20]. By applying tensile stresses, an extended-chain structure locally appears in the strain-hardening stage under uniaxial tension. Thus, in order to achieve a higher oriented state, additional drawing is required. Usually, it is carried out by increasing the drawing temperature. In other words, the slip of the UHMWPE macromolecules will become easy, so the extension of the UHMWPE chains will be significant by increasing the drawing temperature. In addition, the distance between the extended chains will be decreased. So, as an outcome of the intra-molecular and inter-molecular interactions, a recrystallization will occur for the UHMWPE chain molecules [21]. Also, if the draw rate is high enough to prevent the chain retraction, this recrystallization can be obtained and it will not be dissipated by the viscous processes [22]. This draw-induced crystallization can be limited only by a local molecular relaxation [23]. On the other hand, since the relaxation time of the UHMWPE molecules is very long because of the presence of the large amount of the entanglements, this slow relaxation dynamic of the UHMWPE will lead to kinetic activation barriers for the nucleation of the crystallization process. This is why the significant extension of the UHMWPE chains occurs by increasing the drawing temperature, because a faster chain relaxation occurs, which by its turn leads to the increase in the draw ratio and in the crystallinity of the oriented UHMWPE films. Therefore, a multi-stages hot orientation process was carried out in this work by using three different thermal orientation regimes, in order to investigate the influence of the PE-wax presence and its concentration on the drawing process. Also to investigate the influence of the drawing temperature on the final draw ratio values of the UHMWPE/PE-wax films. These three thermal drawing regimes are graphically shown in the Figure 2. Melting temperature has a direct proportional relationship with the molecular weight, only when polyethylene has a molecular weight lower than 10^6^ g/mol [24]. Since the PE-wax has a significant low molecular weight, i.e., low intermolecular bonding forces compared to the UHMWPE; this means that the melting temperature of the PE-wax is lower than the UHMWPE, so the melting temperature of the UHMWPE/PE-wax blends will decrease by the increase of the PE-wax content up to 20.0 wt.%. Therefore, in the first thermal regime, and as it can be seen in Figure 2, the maximum drawing temperature for the UHMWPE/PE-wax films, where the PE-wax content was up to 20.0 wt.%, was 140 °C.

On the other hand, when the PE-wax content in the UHMWPE/PE-wax blends is lower than 2.0 wt.%, the melting temperature of these blends is close to the UHMWPE melting temperature (142.3 °C), which will provide the possibility to increase the drawing temperature up to 142 °C. This is the reason why, in the second thermal regime, the maximum drawing temperature for the UHMWPE/PE-wax films with PE-wax content in the range of 0.1–2.0 wt.% was increased up to 142 °C; in comparison with the first one. 

By taking into consideration, that the presence of the PE-wax as an intermolecular lubricant can reduce the viscosity of the UHMWPE/PE-wax blends, which can provide the possibility to apply harder tensile stresses on the films during orientation process, in order to obtain higher draw ratio; the third thermal regime was applied in order to investigate the influence of the PE-wax presence on the tensile stresses applied on the UHMWPE/PE-wax films. As can be seen in Figure 2, the tensile stresses applied on the films in the third thermal regime are considered harder than the tensile stresses that applied in the second one, especially at the temperatures of 130 °C and above. 

Depending on the PE-wax content, and as can be seen in Figure 2, the maximum draw ratio for each UHMWPE/PE-wax composite for the first thermal regime was in the range of 20–47 at a drawing temperature of 140 °C, while it was in the range of 30–33 and 33–44 consequently for the second and third thermal regimes at a drawing temperature of 142 °C. The final draw ratio values for each UHMWPE/PE-wax composite are mentioned in Table 1.

### 2.3. Testing Procedures

The tensile tests for the oriented UHMWPE/PE-wax films were carried out according to ASTM D882-10 by using Zwick/Roell Z020 universal testing machine (Zwick Roell Group, Ulm, Germany) at 10 mm/min loading rate. At least, 5 samples were measured for each UHMWPE/PE-wax composite. Before doing the test, the surfaces of the films were cleaned by acetone to remove the silicone oil remaining from the drawing step. The UHMWPE films have a low value of friction coefficient, which makes it difficult to do the mechanical tests. Films may slip out of the grips of the testing machine during the test. The increase of the clamping pressure of the grips might lead to a premature failure in the grip area. Therefore, prior to the mechanical tests, both ends of the film were glued to thin cardboards with a size of 60 mm × 50 mm. The tests of the films were carried out using clamping jaws with thin notches, to ensure their reliable fastening for the films without creating local stress concentrations in the capture areas. Slips of the samples during the test were not observed.

SEM micrographs for the structure of the UHMWPE/PE-wax xerogels and films were obtained by using scanning electron microscope JEOL JSM-6610LV at accelerating voltage of 20 kV. The sample surface was coated with a carbon layer of 10–20 nm in thickness (magnetron deposition equipment JFC-1600, JEOL Ltd, Tokyo, Japan was used) in order to avoid a charge accumulation. At least, 3 samples for each UHMWPE/PE-wax composite were studied. After being stored for 30 min in a liquid nitrogen (T = 77 K), the samples of the xerogels were broken into two halves in order to obtain a quasi-brittle fracture surface. The lateral surface of the oriented structure of the UHMWPE/PE-wax films was obtained by the tearing mechanism in the orientation direction. 

Differential scanning calorimetry (DSC) measurements were carried out by using differential scanning calorimetry (NETZSCH DSC 204 F1, NETZSCH Group, Selb, Germany) according to ASTM D 3417-83. A heating rate of 10 °C min^−1^ was applied at a temperature range from 35 to 180 °C. By analyzing the DSC curves, T_m_^onset^ (the onset of the melting peak), T_m_ (the melting temperature peak) and T_m_^end^ (the end of the melting peak) were determined. The relative degree of crystallinity was calculated as the ratio of the experimental sample melting enthalpy to the completely crystallized polyethylene melting enthalpy, which is equal to 293 J/g [25]. The average values of the three samples for each UHMWPE/PE-wax composite were used. The standard deviation for the temperature values was ± 0.1 °C.

Tribological tests were carried out by using Tribometer-CETR-UMT-3 (Bruker Corporation, Karlsruhe, Germany) according to ASTM G 99–95a. Tests were carried out in a dry friction mode in a friction pair: pin on disk, normal loading force 30 N, linear velocity 1 m/s. Friction coefficient was determined after traversing the path (L) of 3.6 km. The 440C stainless steel counter-body with a diameter of 62 mm was used. The counter-body was polished with diamond lapping paste after each test; grits are in the range of 40–60 microns. Films samples with a length of 15 mm and a width of 2.5–3 mm were fixed to a metallic cylinder with a diameter of 10 mm. The metallic cylinder with the fixed sample was pressed against to the rotating steel counter-body. Samples were tested in a parallel direction to the UHMWPE chain orientation. At least, 5 samples for each UHMWPE/PE-wax composite were tested.

## 3. Results and Discussion

Table 1 shows the results of the mechanical tensile tests for the virgin UHMWPE and UHMWPE/PE-wax films obtained by all thermal regimes. It was found that the tensile mechanical properties increased by adding the PE-wax, in comparison with the virgin UHMWPE films. As it can be seen, in Table 1, by applying the second thermal regime on the virgin UHMWPE films, the tensile strength was 743 MPa, the Young’s modulus value was 33.2 GPa and the elongation at break was 4.2%. Also, it was noted that the virgin UHMWPE films had broken off at a temperature of 130 °C, when the third thermal regime was applied because the tensile stresses applied on the films were considered hard, thus these samples were ignored. In addition, the virgin UHMWPE films obtained by the first thermal regime had broken off at a low draw ratio, so these samples were also ignored. As reference values, for the comparison with the mechanical properties of the UHMWPE/PE-wax films, the mechanical properties values of the virgin UHMWPE films obtained by the second thermal regime were used.

For the first thermal regime (Table 1), the tensile strength increased by 78%, in comparison with the virgin UHMWPE films, and peaked (1320 MPa) at a PE-wax content of 1 wt.%. Also, it can be seen that the Young’s modulus values of the UHMWPE/PE-wax films had not changed, in comparison with the virgin UHMWPE film. The elongation at break for all UHMWPE/PE-wax films was in the range of 3.6–7.35%.

For the second thermal regime (Table 1), the tensile strength had increased by 74%, in comparison with the virgin UHMWPE films, and peaked (1290 MPa) at a PE-wax content of 0.1 wt.%. The maximum Young’s modulus value (55.2 GPa) was detected at a PE-wax content of 1.0% (an increase of 66%). The elongation at break for all UHMWPE/PE-wax films was in the range of 3.1–5.5%. It should be noted that the mechanical results for the UHMWPE/PE-wax films with PE-wax content higher than 1.5 wt.% were ignored because the mechanical properties had not improved by using this thermal regime.

By applying the third thermal regime (Table 1), the maximum tensile strength value was 1210 MPa and detected at a PE-wax content of 1 wt.% (an increase of 63%), and the maximum Young’s modulus value (56.8 GPa) was detected at a PE-wax content of 2 wt.% (an increase of 71 %). The elongation at break for all UHMWPE/PE-wax films was in the range of 3.3–5.2%. Also, as it can be seen in Table 1, the addition of the PE-wax provided the possibility to apply higher tensile drawing stresses when the PE-wax content was in the range of 0.5–1.5 wt.%, which led to an increase in the obtained draw ratio values, in comparison with the second thermal regime for each UHMWPE/PE-wax composite at a drawing temperature of 142 °C. It should be mentioned that UHMWPE/PE-wax films with a PE-wax content lower than 0.5 wt.% had broken off at a temperature of 130 °C.

Depending on Table 1, it was found that the presence of the PE-wax had no effect on the final draw ratio values of the UHMWPE/PE-wax films when the PE-wax content was in the range of 0.1–2.0 wt.%. The maximum draw ratio (λ_max_) has linearly inversely proportional relationship with the molecular weight. λ_max_ is depending on the molecular weight of the UHMWPE/PE-wax blends (M_w_) and on the weight fraction of UHMWPE in the blends (φ) as follows [26]:

λ_max_ ~ (φ. M_w_)^-α^ (1)

where α is a power-law coefficient, the value of which depends on the solvent type and the crystallization conditions, and it equals to 0.5 for xylene [26,27]. 

The molecular weight of the UHMWPE/PE-wax blends can be calculated by the following equation [28]:

M_w_ = φ M_w_^UHMWPE^ + (1 − φ) M_w_^PE-wax^ (2)

Where: M_w_^UHMWPE^ and M_w_^PE-wax^ are the molecular weight of the UHMWPE and the PE-wax, respectively.

As can be seen in Equation (2), the relationship between the M_w_ and φ is linear, and the M_w_ decreases linearly by decreasing the φ value. When the PE-wax content is in the range of 0.1–2.0 wt.%, the M_w_ will not decrease practically. So, depending on Equation (1), the maximum draw ratio of the UHMWPE/PE-wax does not depend on the PE-wax content. On the other hand, the final draw ratio values for the UHMWPE/PE-wax films have increased by raising the PE-wax content, leading to a decrease in the M_w_ that has reached 47 at a PE-content of 10.0 wt.% and at a drawing temperature of 140 °C. However, it was found that the maximum draw ratio decreased when the PE-wax content was 20.0 wt.%. It could be explained that the intermolecular bonding forces of the PE-wax were very weak at a drawing temperature of 140 °C. Because of the high amount of the PE-wax located in the UHMWPE amorphous phase, when the PE-wax content was high (20.0 wt.%), the films had broken off in these weak places during the orientation process at a low draw ratio; compared to the UHMWPE/PE-wax films with a PE-wax content of 10.0 wt.%.

One of the important properties of the UHMWPE films for applications is the work of fracture, which presents the ability of the materials to resist fracture. Work of fracture indicates to the material’s resistance to brittle fracture, which means that when the work of fracture is higher, the absorbed energy for rupturing will be higher [29,30]; i.e., the material has a high strength and a high ductility. As it can be seen, in Table 1, the work of fracture for the UHMWPE/PE-wax films was higher, compared to the virgin UHMWPE films, i.e., the UHMWPE/PE-wax films had a higher toughness in comparison with the virgin UHMWPE films. The maximum work of fracture was 106.6 MJ/m^3^, obtained for the UHMWPE/PE-wax films with a PE-wax content of 1.0 wt.% by using the first thermal regime. For example, the work of fracture of the UHMWPE Dyneema fiber is in the range of 45–70 MJ/m^3^ [31]. Thus, the work of fracture of the UHMWPE/PE-wax films is close to the Dyneema fibers.

**Figure 3:**
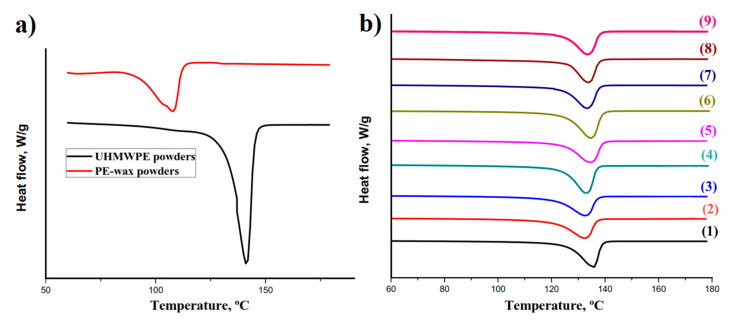
DSC curves for the UHMWPE/PE-wax: (**a**) powders, (**b**) xerogels with PE-wax contents: (1) 0% (virgin UHMWPE), (2) 0.1%, (3) 0.2%, (4) 0.5%, (5) 1.0%, (6) 1.5%, (7) 2.0%, (8) 10.0% and (9) 20.0%.

**Figure 4:**
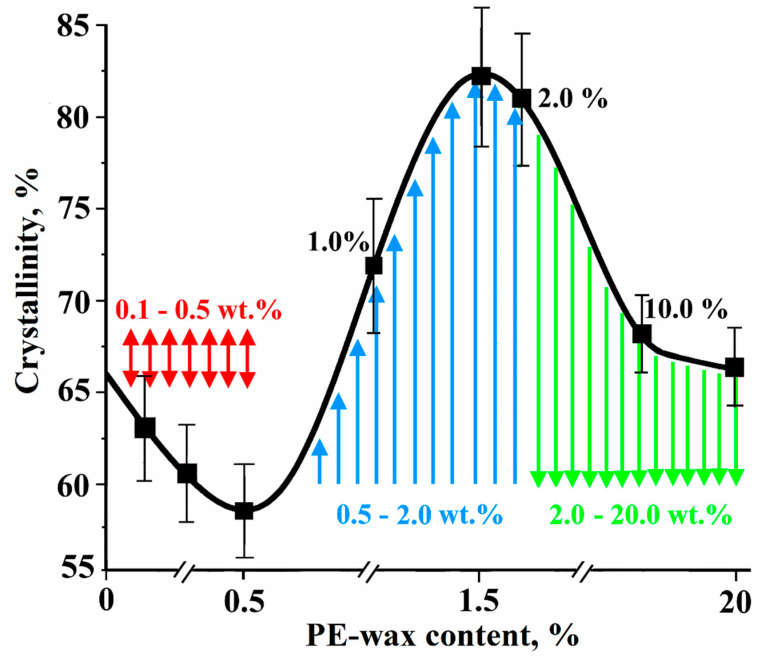
Crystallinity of UHMWPE/PE-wax xerogels depending on PE-wax content.

The results of the DSC test for the polyethylene powders and the UHMWPE/PE-wax xerogels are presented in Table 2 and Table 3 respectively. The DSC heating curves in Figure 3b show that there were not two separate endothermic peaks for the UHMWPE and the PE-wax, which means that the PE-wax had not crystallized separately from the UHMWPE. Therefore, the PE-wax has a good miscibility with the UHMWPE. Figure 4 shows the crystallinity curve depending on the PE-wax content for the UHMWPE/PE-wax xerogels. As can be seen in Table 3 and Figure 4, when the PE-wax content was in the range of 0.1–0.5 wt.%, the crystallinity of the UHMWPE/PE-wax xerogels was in the range of standard deviation values of the crystallinity of the virgin UHMWPE xerogel; which means that practically the PE-wax had no effect on the crystallinity of the UHMWPE/PE-wax xerogels in this range of the PE-wax content. On the other hand, when the PE-wax content was in the range of 0.5–2.0 wt.%, the crystallinity of UHMWPE/PE-wax xerogels was increased up to 82%, in comparison with the virgin UHMWPE xerogels. It could be clarified by the significant low molecular weight of the PE-wax that has a noticeable low melting temperature, in comparison with the UHMWPE. So that the presence of the PE-wax as an intermolecular lubricant will reduce the viscosity of the UHMWPE/PE-wax blends, and this will make the UHMWPE chains move easily, i.e., the presence of the PE-wax leads to an intensive slippage of the UHMWPE molecular. In other words, the UHMWPE amorphous phase, where the entanglements are located, will be narrower, which will increase the crystallinity [32,33]. Also, as it can be seen, in Table 3 and Figure 4, when the PE-wax content was in the range of 10.0–20.0 wt.%, the crystallinity of the UHMWPE/PE-wax xerogels decreased to standard deviation values of the crystallinity of the virgin UHMWPE xerogel. This is because the PE-wax has a low crystallinity (54%), so when the PE-wax content was increased up to 20.0 wt.%, nothing changed in its original state in a large amount of it, which led to a decrease in the crystallinity of the UHMWPE/PE-wax xerogels. 

In addition, as can be seen in Table 3, the presence of the PE-wax led to a decrease in the melting temperature of the UHMWPE/PE-wax xerogels, in comparison with the virgin UHMWPE xerogel. This decrease in the melting temperature is probably related to the role of the PE-wax as an intermolecular lubricant, which causes thermo-dynamical and morphological changes in the amorphous phase nature and the lamellar thickness of the UHMWPE [34]. On the other hand, when the PE-wax content increased higher than 0.5 wt.% (in the range of 1.0–20.0 wt.%), an increase in the values of the crystallinity and the melting temperature of the UHMWPE/PE-wax xerogels was noted. 

The results of the DSC test for the films, obtained by all thermal regimes, are presented in Table 4. As can be seen in Table 4, the UHMWPE/PE-wax films, obtained by all thermal regimes, had a high crystallinity in the range between 84% and 98% compared to the virgin UHMWPE films (77%), as a result of the orientation of the UHMWPE amorphous phase and the deployment of lamellae to the drawing direction and their recrystallization, which were proceeding simultaneously with a reduction of the crystalline phase defects. Also, compared to the UHMWPE/PE-wax xerogels, the melting temperature of the UHMWPE/PE-wax films increased (Table 3 and Table 4), and this means a formation of the extended-chain crystals, which have a higher melting point. In other words, the folded-chain lamella transformed into oriented extended-chain crystals (fibrillar structure) as a result of the drawing process [35]. According to the Thomson–Gibbs equation [36], the melting temperature of polymers depends mainly on the crystalline phase size, i.e., a larger size and non-defected crystallites have a higher melting temperature in comparison with small and defected crystallites. During the orientation process, the density and the number of chains holding the load increases, and the amorphous phase will undergo a deformation during elongation, which leads to an increase in the melting temperature and the crystallinity of the oriented films. 

It should be noted that, with the addition of the PE-wax provided the possibility to apply high tensile drawing stresses for each UHMWPE/PE-wax composite, as proven above in Table 1, the effect of the increase in the draw ratio values among the UHMWPE/PE-wax films, obtained by the second and the third thermal regimes, on their melting temperature values are clearly shown in Table 4. As can be seen in Table 1 and Table 4, the melting temperature of the UHMWPE/PE-wax films obtained by the third thermal regime increased when the maximum draw ratio increased for each UHMWPE/PE-wax composite, in comparison with the UHMWPE/PE-wax films obtained by the second thermal regime.

**Figure 5:**
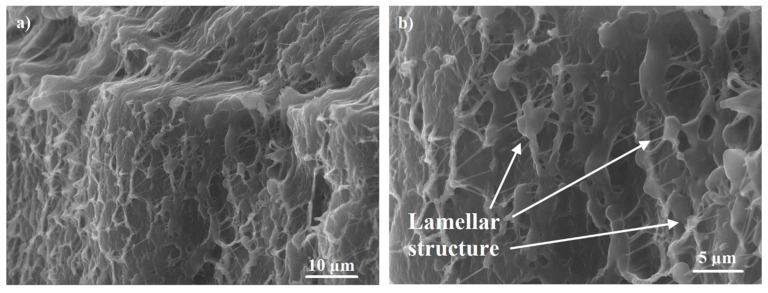
SEM micrographs showing typical supra-molecular structure of the virgin UHMWPE xerogel with different magnification.

**Figure 6:**
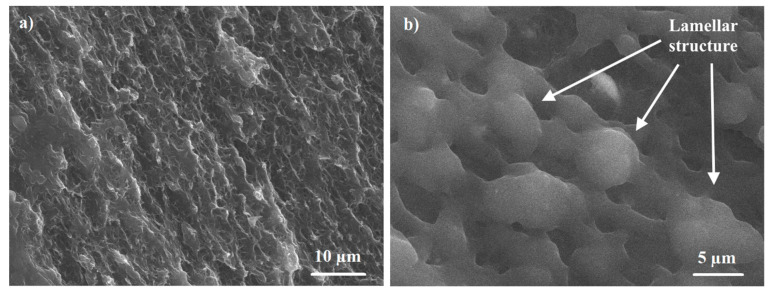
SEM micrographs showing typical supra-molecular structure of the UHMWPE/PE-wax xerogel with different magnification.

Figure 5 and Figure 6 show the typical supra-molecular structure for the virgin UHMWPE and the UHMWPE/PE-wax xerogels, respectively. The supra-molecular structure of the virgin UHMWPE and the UHMWPE/PE-wax xerogels is a lamellar crystalline structure, which exhibits a high processability. 

**Figure 7:**
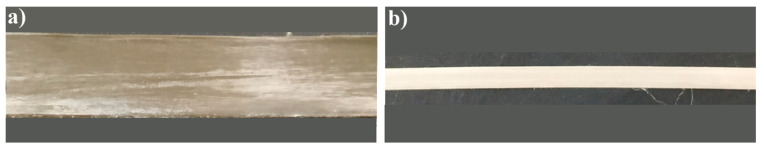
UHMWPE films form before (**a**) and after (**b**) drawing process.

The orientation process of the UHMWPE films leads to a change in the final color of the obtained films. This change in color is called the whitening process (Figure 7). The change in the final color of the oriented films can be related to the crystallization process and cavitation phenomenon. In the tensile deformation process, the lamellar structure will be reformed into a highly oriented fibrillar structure as a result of the fibrillation process. A further tensile deformation will lead to a stretching of the formed fibrillar structure, which by its turn will lead to a decrease in the thickness of the main fibrils (a group of micro/nano fibrils) and a rupture of the highly oriented amorphous network, which was built up by the load-bearing inter-fibril tie chains, causing the formation of cavities [37].

In general, if the polymer has a high entanglement density, the formation of the cavitation is considered difficult [38]. Cavitation phenomenon can be observed in many semi-crystalline polymers like UHMWPE during tensile deformation at temperatures above the glass transition temperature. Cavitation seems around the yield point and only in the tension state, and it will not occur during compression or shearing [39,40,41,42]. The appearance of cavities in the structure of the oriented films leads to a reduction in the obtained mechanical properties. In addition, the maximum formation of cavities occurs at the yield point. Thus, if the cavitation occurs at the beginning of the drawing process, this will lead to a premature destruction of the films, which means that the optimal degree of the draw ratio cannot be achieved. Therefore, it is very important to avoid the formation of cavities at the initial stage of the drawing process by using the rolling process. So, when the films are rolled at the first stage of the orientation process at 110 °C to reach a draw ratio of 1.5–2.0, the voids will not appear in this stage, because the films were deformed by compressive stresses. 

**Figure 8:**
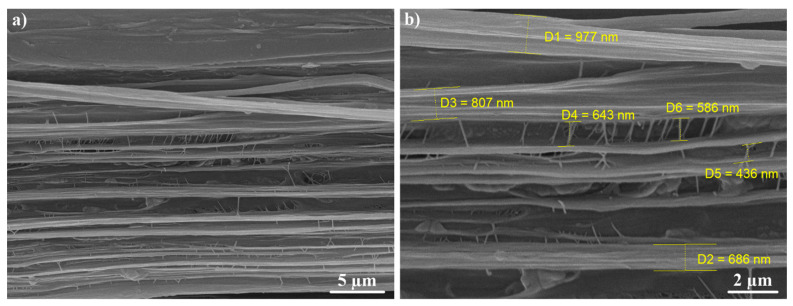
SEM images of fibrillar structure with different magnifications for virgin UHMWPE films. Tensile direction is horizontal.

**Figure 9:**
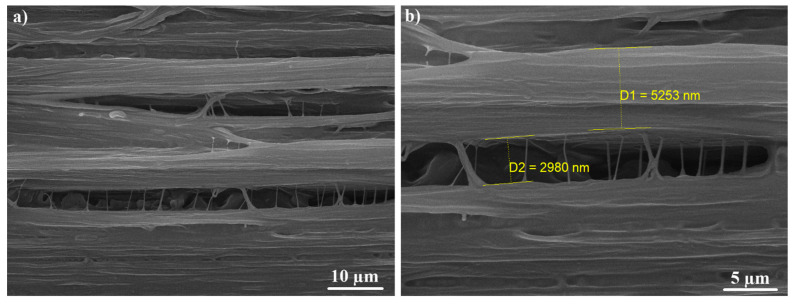
SEM images of fibrillar structure with different magnifications for UHMWPE/PE-wax films at a PE-wax content of 1.0 wt.% with a draw ratio value of 33 obtained by second thermal regime. Tensile direction is horizontal.

**Figure 10:**
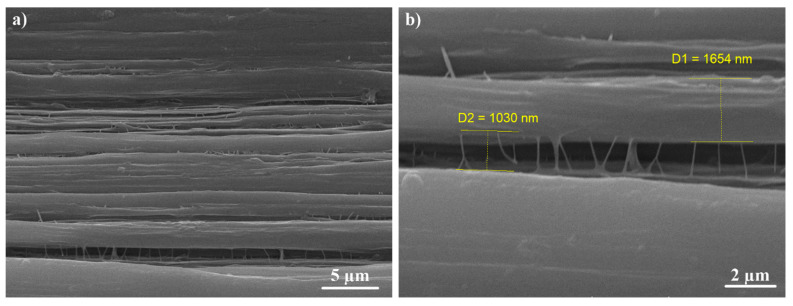
SEM images of fibrillar structure with different magnifications for UHMWPE/PE-wax films at a PE-wax content of 1.0 wt.% with a draw ratio value of 23 obtained by first thermal regime. Tensile direction is horizontal.

Figure 8, Figure 9 and Figure 10 show the fibrillar structure of the virgin UHMWPE and the UHMWPE/PE-wax films. As it can be seen, in Figure 8, the average thickness of main fibrils in the fibrillar structure of the virgin UHMWPE films is about 800 nm, and the average length of the tie fibrils is about 555 nm. Also, as it can be seen, in Figure 9 and Figure 10, the average thickness of main fibrils in the fibrillar structure of the UHMWPE/PE-wax films at a PE-wax content of 1.0 wt.% with draw ratio values of 33 and 23 is about 5.2 µm and 1.7 µm, respectively; and the average length of the tie fibrils is about 3 µm and 1 µm, respectively. So, the presence of the PE-wax led to the increase of the thickness of main fibrils and the length of the tie fibrils from nano size for the virgin UHMWPE films to micro size for the UHMWPE/PE-wax films. Also, when the draw ratio value of the UHMWPE/PE-wax films increased, the thickness of the main fibrils and the length of the tie fibrils increased. As a result, the formation of the cavities occurred in the virgin UHMWPE films more than in the UHMWPE/PE-wax films.

**Figure 11:**
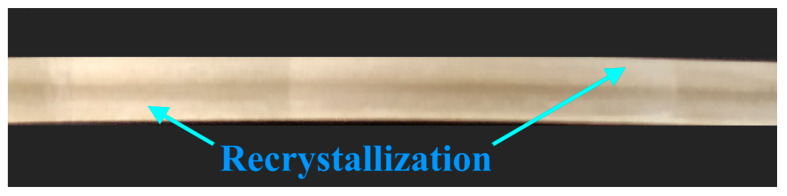
Color change (whitening) of UHMWPE-PE-wax films related to recrystallization process.

As it was mentioned above in the materials and methods section, if draw rate is high enough to prevent the chain retraction, the recrystallization process will occur for the UHMWPE chain molecules and it will not be dissipated by the viscous processes. In other words, the recrystallization process will occur as an outcome of the intra-molecular and inter-molecular interactions during the orientation process [1]. When the extrusion molding is used to prepare the polymer films, the high shear regions will be near the xerogel walls, which mean that the presence of the oriented layers will be higher in the wall area than in the center area of the xerogel [21,23]. Hence, when the first and the second thermal regimes were applied on the films, i.e., in the case that the applied tensile stresses were soft, the recrystallization began to be visible at a temperature of 140 °C. On the other hand, when the third thermal regime was applied on the films, i.e., in the case that the applied tensile stresses were harder, in comparison with the first and second thermal regimes, especially at a temperature of 130 °C, the recrystallization began to be visible early at a temperature of 135 °C. As can be seen, in Figure 11, the recrystallization became visible near the walls of the films, where the presence of the oriented layers was higher, compared to the center of the films, then it grew towards the center, where the regions had low stresses, i.e., less presence of the oriented layers. Lin et al. reported that thin cavities will appear when the drawing temperature value is lower than the onset-temperature value (the temperature at the beginning of the melting peak), while when the drawing temperature value is higher than the onset-temperature, no cavities will be obtained after drawing [43]. As a result, these visible changes in the color of the films occurred at a drawing temperature higher than the onset-temperature, which means that the color change of the films here was related to the recrystallization process more than to the cavitation phenomenon. 

The orientation process for the UHMWPE structure has an important effect not only on the mechanical properties, but also on the tribological properties. When the UHMWPE chains are oriented, the thickness of the lamellae will be decreased, and this leads to an increase in the wear resistance of the material. Also, the friction film thickness is thinner in the case of the oriented UHMWPE films [44]. 

**Figure 12:**
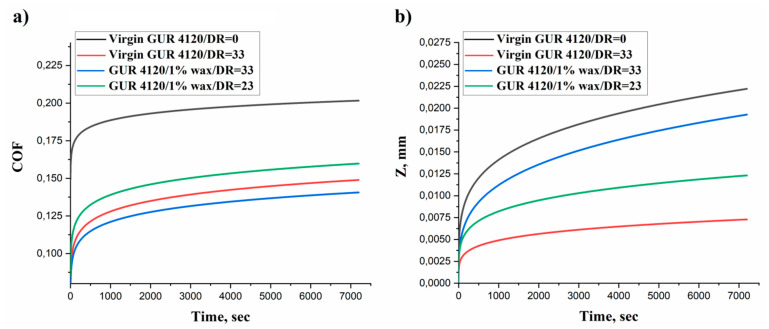
Coefficient of friction (**a**) and wear (**b**) curves for isotropic UHMWPE and virgin UHMWPE film in comparison with UHMWPE/PE-wax films with different draw ratio values.

Table 5 shows the tribological properties of the isotropic UHMWPE, the virgin UHMWPE film and the UHMWPE/PE-wax films with different draw ratio values. The UHMWPE/PE-wax film obtained by the first thermal regime with PE-wax content of 1.0 wt.% and λ = 23 was chosen, because it had the maximum tensile strength and work of fracture values; and in order to investigate the influence of the draw ratio values on the tribological properties, the UHMWPE/PE-wax film obtained by the second thermal regime with PE-wax content of 1.0 wt.% and λ = 33 was chosen. The tribological properties values were recorded after two hours of friction (3.6 km) and under a load of 30 N. Figure 12 shows the coefficient of friction (COF) and wear curves for the isotropic UHMWPE, the virgin UHMWPE film and the UHMWPE/PE-wax films with different draw ratio values. COF value for isotropic UHMWPE is generally low. So, what should be taken into consideration is that the increase in the applied load will lead to the increase of the COF value, since this value has a significant dependence on the applied load values [45]. Therefore, in order to investigate the effect of the orientation process on the COF values of the oriented films, the applied load in this study is considered heavy (30 N), which equals about 4 MPa as a contact pressure (contact pressure is the ratio of the applied load to the area of the wear spot at the end of the test). 

As it can be seen in Table 5 and Figure 12, the oriented films had better tribological properties in comparison with the isotropic UHMWPE. Also, the highly oriented UHMWPE/PE-wax films with a DR = 33 had the best COF value (0.14). In comparison with the polytetrafluoroethylene-based composites (PTFE, Teflon), which is considered a popular material used in a large number of tribological applications due to its low COF value, ranges between 0.05 and 0.20 depending on the load, sliding speed, and coating [46]; the oriented UHMWPE/PE-wax films had a COF value similar to PTFE.

When the mechanical properties are enhanced by the orientation process, i.e., the improvement of the tensile strength and Young’s modulus, and the increase of the oriented films crystallinity; the wear behavior will improve [47,48]. Accordingly, the hardness that refers to the material’s resistance to the surface deformation will increase as a result of the orientation process. This means that the enhancement in the rigidity will lead to a decrease in the deformation volume of the friction surface, that occurs on the films surface, i.e., the possibility of the penetration of the counter-body into the friction surface of the films will decrease, leading to a significant decrease in the dry COF values of the oriented films, in comparison with the isotropic materials. In other words, the increase in the hardness and rigidity of the oriented UHMWPE films enable the application of high contact pressure and preserving good antifriction properties.

However, the PE-wax presence, with its effect as a lubricant, can lead to a reduction in the wear resistance of the films [17]. Consequently, when the mechanical properties of the UHMWPE/PE-wax films are better and their crystallinity is higher (UHMWPE/PE-wax films with PE-wax content of 1.0 wt.% and λ = 33), in comparison with the virgin UHMWPE films, the coefficient of friction will be low, although the wear resistance will be lower than before. On the other hand, for the UHMWPE/PE-wax films with a PE-wax content of 1.0 wt.% and draw ratio of 23, which have better mechanical properties but lower crystallinity compared to the UHMWPE/PE-wax films with PE-wax content of 1.0 wt.% and draw ratio of 33, the presence of the PE-wax here plays an important role as a lubricant, leading to a higher wear resistance, though the coefficient of friction value will also be higher.

## 4. Conclusions 

In this paper, the PE-wax was added to the UHMWPE in a small amount as an intermolecular lubricant to improve the processability of the lamellar structure and the mechanical properties of the highly oriented UHMWPE/PE-wax films. As evidenced by DSC and SEM, the PE-wax had a good miscibility with the UHMWPE and it had dispersed uniformly in the UHMWPE matrix. What was found is that when the PE-wax content was in the range of 0.5–2.0 wt.%, the crystallinity of the UHMWPE/PE-wax xerogels increased. The reason to that was the role of the PE-wax as an intermolecular lubricant, which reduced the viscosity of the UHMWPE/PE-wax blends and facilitated the movements of the UHMWPE chains, i.e., the UHMWPE amorphous phase, where the entanglements are located, became narrower, and the crystallinity increased. On the other hand, when the PE-wax content was in the range of 2.0–20.0 wt.%, the crystallinity of the UHMWPE/PE-wax xerogels decreased due to the presence of a large amount of the PE-wax that preserved its original state as a PE with a low crystallinity. In addition, it was found that the presence of the PE-wax led to a decrease in the melting temperature of the UHMWPE/PE-wax xerogels, in comparison with the virgin UHMWPE xerogel due to the role of the PE-wax as an intermolecular lubricant, which causes thermo-dynamical and morphological changes in the amorphous phase nature and the lamellar thickness of the UHMWPE. Furthermore, it was found that UHMWPE/PE-wax films had a high crystallinity, in the range between 84% and 98% in comparison with the virgin UHMWPE films, as a result of the orientation of the UHMWPE amorphous phase and the deployment of lamellae to the drawing direction and their recrystallization. In addition, the melting peak of the highly oriented UHMWPE/PE-wax films increased, in comparison with the UHMWPE/PE-wax xerogels, which means that the folded-chain lamella transformed into oriented extended-chain crystals as a result of the drawing process.

The PE-wax presence greatly enhanced the mechanical properties of the UHMWPE/PE-wax films. The highest average value of the tensile strength was 1320 MPa (an increase of 78%), obtained with a PE-wax content of 1.0 wt.%, and the highest value of the Young’s modulus was 56.8 GPa (an increase of 71%), obtained with a PE-wax content of 2.0 wt.%. When the PE-wax content was in the range of 0.1–2.0 wt.%, in comparison with the virgin UHMWPE films, there was no effect on the final draw ratio values of the UHMWPE/PE-wax films; and when it was in the range of 10.0–20.0 wt.%, there was not any noticeable influence of the addition of the PE-wax on the mechanical properties of the UHMWPE/PE-wax films, in comparison with the virgin UHMWPE films. Moreover, when the PE-wax content was in the range of 0.5–1.5 wt.%, the addition of it allowed for the application of high tensile drawing stresses. It was found that the work of fracture of the UHMWPE/PE-wax films improved up to 233%, in comparison with the virgin UHMWPE films. The maximum work of fracture was 106.6 MJ/m^3^ obtained for the UHMWPE/PE-wax films with a PE-wax content of 1.0 wt.%.

The presence of the PE-wax increased the thickness of main fibrils and the length of the tie fibrils in the fibrillar structure from nano size for the virgin UHMWPE films to micro size for the UHMWPE/PE-wax films. Also, when the draw ratio value of the UHMWPE/PE-wax films increased, the thickness of the main fibrils and the length of the tie fibrils increased. As a result, the formation of the cavities that occurred in the virgin UHMWPE films was more than the one occurred in the UHMWPE/PE-wax films and the visible changes in the color of the films occurred at a drawing temperature that was higher than the onset-temperature value, which means that the whitening of the oriented UHMWPE/PE-wax films was related to the crystallization process more than to the cavitation phenomenon.

The oriented films had better tribological properties in comparison with the isotropic UHMWPE. The COF value of the oriented UHMWPE/PE-wax films with a PE-wax content of 1.0 wt.% and a draw ratio value of 33 was better than the COF value of the isotropic UHMWPE and the oriented virgin UHMWPE films by 33% and 7% respectively. In contrast, the wear resistance of the virgin UHMWPE films was better than the wear resistance of the UHMWPE/PE-wax films due to the PE-wax effect as a lubricant.

Finally, since these highly oriented and strengthened films are fabricated from cheap large-scale brands of UHMWPE, their production cost is lower than the ones fabricated from specific brands of the UHMWPE reactor powder. Therefore, the UHMWPE/PE-wax films obtained by using a continuous process are considered the best choice for both biomedical and industrial applications, especially for the tribological applications.

## Figures and Tables

**Table 1:** Mechanical tensile properties of the UHMWPE/PE-wax films with different PE-wax content for all thermal regimes (± the standard deviation).

Material	Thermal Regime Type	Wax Content, %	Final DR	Young’s Modulus, GPa	Tensile Strength, MPa	Elongation, %	Work of Fracture, MJ/m^3^
**Virgin UHMWPE Films**	**Second Thermal Regime**	0	33	33.2 ± 2.6	743 ± 68	4.20 ± 0.35	31.98 ± 5.1
**UHMWPE/PE-Wax Films**	**First Thermal Regime**	0.1	20	26.2 ± 1.2	1080 ± 52	5.61 ± 1.1	70.50 ± 6.3
		0.2	20	26.0 ±1.7	1090 ± 59	5.90 ± 1.0	70.69 ± 6.8
		0.5	23	30.6 ± 4.7	963 ± 117	4.65 ± 0.7	51.8 ± 4.8
		1.0	23	31.6 ± 2.0	1320 ± 31	7.35 ± 1.4	106.6 ± 10.1
		2.0	25	30.8 ± 2.3	1110 ± 82	5.55 ± 1.9	72.46 ± 6.8
		10.0	47	34.2 ± 0.7	883 ± 18	3.6 ± 0.3	34.62 ± 2.8
		20.0	30	33.2 ± 3.3	866 ± 59	6.3 ± 0.55	63.07 ± 6.4
**UHMWPE/PE-Wax Films**	**Second Thermal Regime**	0.1	30	44.8 ± 2.8	1290 ± 59	5.25 ± 0.75	71.82 ± 6.1
		0.2	30	44.8 ± 6.1	1260 ± 109	5.50 ± 0.45	72.63 ± 6.2
		0.5	33	35.0 ± 4.8	1080 ± 134	4.60 ± 0.90	67.45 ± 7.1
		1.0	33	55.2 ± 3.5	1080 ± 104	3.1 ± 0.40	58.23 ± 5.3
		1.5	30	51.2 ± 3.7	1100 ± 37	3.75 ± 0.45	45.92 ± 6.2
**UHMWPE/PE-Wax Films**	**Third Thermal Regime**	0.5	44	48.8 ± 3.5	1130 ± 76	4.0 ± 0.45	51.30 ± 5.5
		1.0	37	44.4 ± 0.1	1210 ± 50	5.2 ± 0.75	75.54 ± 5.6
		1.5	37	52.0 ± 6.4	1120 ± 105	3.3 ± 0.75	57.58 ± 5.1
		2.0	33	56.8 ± 3.1	1150 ± 81	3.3 ± 0.45	46.30 ± 5.0

**Table 2:** DSC test results for the UHMWPE and PE-wax powders (± the standard deviation).

Material	T_m_^onset^, °C	T_m_, °C	T_m_^end^, °C	Crystallinity, %
**UHMWPE**	132.6	142.3	145.7	67 ± 2
**PE-wax**	75.0	108.5	112.1	54 ± 2

**Table 3:** DSC test results for the UHMWPE/PE-wax xerogels depending on PE-wax content (± the standard deviation).

Material	PE-wax Content, %	T_m_^onset^, °C	T_m_, °C	T_m_^end^, °C	Crystallinity, %
**UHMWPE/PE-wax xerogels**	0	126.4	135.8	138.9	66 ± 2
	0.1	122.7	133.0	136.7	60 ± 2
	0.2	121.7	132.2	136.7	59 ± 3
	0.5	123.4	132.6	136.3	60 ± 2
	1.0	125.4	134.7	139.9	72 ± 5
	1.5	125.6	134.6	139.0	82 ± 5
	2.0	126.6	135.7	139.9	78 ± 4
	10.0	126.0	134.2	138.7	68 ± 3
	20.0	128.4	135.7	139.6	66 ± 2

**Table 4:** DSC test results for the UHMWPE/PE-wax films depending on the PE-wax content for all thermal regimes (± the standard deviation).

Material	Thermal Regime Type	PE-wax Content, %	T_m_^onset^, °C	T_m_, °C	T_m_^end^, °C	Crystallinity, %
**Virgin UHMWPE films**	**Second Thermal Regime**	0	139.5	142.1	144.3	77 ± 2
**UHMWPE/PE-wax Films**	**First Thermal Regime**	0.1	136.5	141.3	145.4	82 ± 3
		0.2	136.7	141.6	145.3	82 ± 2
		0.5	139.0	142.4	145.6	85 ± 2
		1.0	138.9	142.4	145.7	87 ± 3
		2.0	139.1	143.6	145.6	86 ± 3
		10.0	138.1	141.7	145.1	85 ± 2
		20.0	136.0	140.5	144.4	75 ± 3
**UHMWPE/PE-wax Films**	**Second Thermal Regime**	0.1	138.5	142.5	145.1	89 ± 2
		0.2	140.0	143.5	145.2	98 ± 2
		0.5	139.0	142.5	146.6	95 ± 2
		1.0	139.3	142.5	144.4	96 ± 2
		1.5	140.2	143.4	145.0	91± 2
**UHMWPE/PE-wax Films**	**Third Thermal Regime**	0.5	139.7	144.2	145.6	90 ± 2
		1.0	139.0	143.3	145.8	95 ± 3
		1.5	139.8	143.5	145.2	97 ± 2
		2.0	139.4	143.9	145.1	84 ± 3

**Table 5:** Tribological properties of isotropic UHMWPE and virgin UHMWPE film in comparison with UHMWPE/1.0 % PE-wax films with different draw ratio values (±the standard deviation).

Material	COF	Z, mm
**Isotropic Virgin UHMWPE**	0.21 ± 0.010	0.022 ± 0.003
**Virgin UHMWPE films - DR=33**	0.15 ± 0.010	0.007 ± 0.002
**UHMWPE/1% PE-wax films- DR=33**	0.14 ± 0.005	0.019 ± 0.003
**UHMWPE/1% PE-wax films- DR=23**	0.16 ± 0.010	0.012 ± 0.003
